# Supplementary material for: The Differences in Clinical Presentation, Management, and Prognosis of Laboratory-Confirmed COVID-19 between Pregnant and Non-Pregnant Women: A Systematic Review and Meta-Analysis
Source: Int J Environ Res Public Health. 2021 May 24;18(11):5613. doi: 10.3390/ijerph18115613 (PMC8197383; doi:10.3390/ijerph18115613)
Supplement: Supplementary file 1 [file ijerph-18-05613-s001.zip › ijerph-1211523-SI.pdf]

Supplementary tables

Supplementary Table S1: PRISMA Checklist

Section/topic	#	Checklist item	Reported on page #
**TITLE**			
Title	1	Identify the report as a systematic review, meta-analysis, or both.	1
**ABSTRACT**			
Structured summary	2	Provide a structured summary including, as applicable: background; objectives; data sources; study eligibility criteria, participants, and interventions; study appraisal and synthesis methods; results; limitations; conclusions and implications of key findings; systematic review registration number.	1
**INTRODUCTION**			
Rationale	3	Describe the rationale for the review in the context of what is already known.	3
Objectives	4	Provide an explicit statement of questions being addressed with reference to participants, interventions, comparisons, outcomes, and study design (PICOS).	3
**METHODS**			

Protocol and registration	5	Indicate if a review protocol exists, if and where it can be accessed (e.g., Web address), and, if available, provide registration information including registration number.	4
Eligibility criteria	6	Specify study characteristics (e.g., PICOS, length of follow-up) and report characteristics (e.g., years considered, language, publication status) used as criteria for eligibility, giving rationale.	4
Information sources	7	Describe all information sources (e.g., databases with dates of coverage, contact with study authors to identify additional studies) in the search and date last searched.	4
Search	8	Present full electronic search strategy for at least one database, including any limits used, such that it could be repeated.	4, 18
Study selection	9	State the process for selecting studies (i.e., screening, eligibility, included in systematic review, and, if applicable, included in the meta-analysis).	4
Data collection process	10	Describe method of data extraction from reports (e.g., piloted forms, independently, in duplicate) and any processes for obtaining and confirming data from investigators.	4, 5
Data items	11	List and define all variables for which data were sought (e.g., PICOS, funding sources) and any assumptions and simplifications made.	4, 5
Risk of bias in individual studies	12	Describe methods used for assessing risk of bias of individual studies (including specification of whether this was done at the study or outcome level), and how this information is to be used in any data synthesis.	5

Summary measures	13	State the principal summary measures (e.g., risk ratio, difference in means).	5
Synthesis of results	14	Describe the methods of handling data and combining results of studies, if done, including measures of consistency (e.g., I^2) for each meta-analysis.	5
Risk of bias across studies	15	Specify any assessment of risk of bias that may affect the cumulative evidence (e.g., publication bias, selective reporting within studies).	NA
Additional analyses	16	Describe methods of additional analyses (e.g., sensitivity or subgroup analyses, meta-regression), if done, indicating which were pre-specified.	NA
**RESULTS**			
Study selection	17	Give numbers of studies screened, assessed for eligibility, and included in the review, with reasons for exclusions at each stage, ideally with a flow diagram.	5, 16
Study characteristics	18	For each study, present characteristics for which data were extracted (e.g., study size, PICOS, follow-up period) and provide the citations.	5
Risk of bias within studies	19	Present data on risk of bias of each study and, if available, any outcome level assessment (see item 12).	15
Results of individual studies	20	For all outcomes considered (benefits or harms), present, for each study: (a) simple summary data for each intervention group (b) effect estimates and confidence intervals, ideally with a forest plot.	5, 6, 17
Synthesis of results	21	Present results of each meta-analysis done, including confidence intervals and measures of consistency.	5, 6, 15

Risk of bias across studies	22	Present results of any assessment of risk of bias across studies (see Item 15).	
Additional analysis	23	Give results of additional analyses, if done (e.g., sensitivity or subgroup analyses, meta-regression [see Item 16]).	NA
**DISCUSSION**			
Summary of evidence	24	Summarize the main findings including the strength of evidence for each main outcome; consider their relevance to key groups (e.g., healthcare providers, users, and policy makers).	7
Limitations	25	Discuss limitations at study and outcome level (e.g., risk of bias), and at review-level (e.g., incomplete retrieval of identified research, reporting bias).	8
Conclusions	26	Provide a general interpretation of the results in the context of other evidence, and implications for future research.	8
**FUNDING**			
Funding	27	Describe sources of funding for the systematic review and other support (e.g., supply of data); role of funders for the systematic review.	1

Supplementary Table S2: Search Strategy

("Symptomatic pregnant" OR ("symptomatic" AND "pregnant") OR "Pregnant" OR "Woman, pregnant" OR "Pregnant Women"[MeSH Terms] OR "Pregnant Woman" OR "Pregnant Women" OR "Pregnancy" OR "Pregnancies" OR "Pregnant" OR "Gravid" OR "Gravidity" OR "Pregnancy"[MeSH Terms] OR "Gestation" OR "mid-pregnancy" OR "Obstetric" OR "Maternal" OR "Pregnant Females" OR "Parturient")<br><br>AND<br><br>("Coronavirus" OR "corona virus" OR "corona virus disease""COVID-19" OR "2019 novel coronavirus disease" OR "COVID19" OR "COVID-19 pandemic" OR "SARS-CoV-2 infection" OR "Severe acute respiratory syndrome coronavirus 2" OR "COVID-19 virus disease" OR "2019 novel coronavirus infection" OR "2019-nCoV infection" OR "coronavirus disease 2019" OR "coronavirus disease-19" OR "2019-nCoV disease" OR "COVID-19 virus infection" OR "corona virus 2019" OR "sars-cov-2" OR "sars-cov2" OR ("wuhan*" AND (("virus" OR "viruses" OR "viral") OR "coronav*")) OR ("covid*" AND ("virus" OR "viruses" OR "viral")) OR "sars-cov-2" OR "sars cov 2" OR "2019nCoV" OR "novel corona virus")<br><br>AND<br><br>("non-pregnant" OR "non-pregnant women" OR "non-pregnant woman" OR "women" OR "woman" OR "asymptomatic" OR "asymptomatic pregnant")<br><br>AND<br><br>("outcomes" OR "symptoms" OR "Signs and Symptoms"[MeSH Terms] OR "Clinical presentation" OR "Clinical Characteristics" OR "Clinical manifestation*" OR "Laboratory test*" OR "Laboratory characteristics" OR "Complications"[Subheading] OR "Prevalence"[MeSH Terms] OR "Treatment*" OR "Morbidity" OR "Mortality" OR "Clinical features" OR "ventilatory support" OR "Antiviral therapy" OR "corticosteroids" OR "oxygen therapy" OR "Chinese medicine" OR "Antibiotics" OR "immunoglobulin" OR "postpartum outcomes" OR "pregnancy outcomes" OR "neonatal outcomes" OR "Comorbidity"[MeSH Terms]) OR "Prognosis" OR "Management")

Supplementary Table S3: Overlapping studies

Overlapping Studies			
**Study**	**Center, Country and Time period**	**Overlap Study**	**Center, Country and Time period**
Laura Zambrano, 2020 (1)	CDC directory via National Notifiable Diseases Surveillance System (NNDSS)<br>Jan 22nd to Oct 3rd, 2020<br>Sample size: 409,462	Asma Tekbali, 2020(4)	14 hospitals<br>NYC, USA<br>Mar 2nd to Mar 29th, 2020<br>Sample Size: 21,980
Shaoshuai Wang, 2020(2)	Tongji Hospital<br>Wuhan, China<br>Jan 19th to Mar 2nd, 2020<br>Sample Size: 43	Ming-Zhu Yin, 2020(5)	Wuhan Union and Tongji hospital<br>Wuhan, China<br>Jan 28 to Feb 28, 2020<br>Sample Size: 66
Xu Qiancheng, 2020(3)	Central Hospital of Wuhan<br>Wuhan, China<br>Jan 15th to Mar 15th, 2020<br>Sample size: 82	Zhiqiang Wang, 2020(6)	Central Hospital of Wuhan<br>Wuhan, China<br>Dec 8th, 2019 to Apr 2020<br>Sample size 72
Laura Zambrano, 2020 (1)	CDC directory via National Notifiable Diseases Surveillance System (NNDSS)<br>Jan 22nd to Oct 3rd, 2020<br>Sample size: 409,462	Sascha Ellington, 2020(7)	CDC directory covering 50 states,<br>District of Columbia and NYC, USA<br>Jan 22nd to Jun 7th, 2020<br>Sample size: 91,412

References for supplementary file:

1. Zambrano LD, Ellington S, Strid P, Galang RR, Oduyebo T, Tong VT, et al. Update: Characteristics of Symptomatic Women of Reproductive Age with Laboratory-Confirmed SARS-CoV-2 Infection by Pregnancy Status — United States, January 22–October 3, 2020. MMWR Morb Mortal Wkly Rep. 2020;
2. Shaoshuai Wang, Lijie Wei, Xuan Gao, Suhua Chen, Wanjiang Zeng JW, Xingguang Lin, Huiting Zhang, Lali Mwamaka Sharifu, Ling Chen LF. Clinical characteristics and outcomes of childbearingage women with Coronavirus disease 2019 in Wuhan: a retrospective, single-center study. JMIR. 2020;
3. Qiancheng X, Jian S, Lingling P, Lei H, Xiaogan J, Weihua L, et al. Coronavirus disease 2019 in pregnancy. Int J Infect Dis. 2020;
4. Tekbali A, Grünebaum A, Saraya A, McCullough L, Bornstein E, Chervenak FA. Pregnant vs nonpregnant severe acute respiratory syndrome coronavirus 2 and coronavirus disease 2019 hospital admissions: the first 4 weeks in New York. Am J Obstet Gynecol [Internet]. 2020 Jul;223(1):126–7. Available from: https://linkinghub.elsevier.com/retrieve/pii/S0002937820304373
5. Yin M-Z. Severe Acute Respiratory Syndrome Coronavirus 2 (SARS-CoV-2) Infection During Pregnancy In China: A Retrospective Cohort Study. 2020;
6. Wang Z, Wang Z, Xiong G. Clinical characteristics and laboratory results of pregnant women with COVID-19 in Wuhan, China. Int J Gynecol Obstet [Internet]. 2020 Sep 3;150(3):312–7. Available from: https://onlinelibrary.wiley.com/doi/abs/10.1002/ijgo.13265

7. Ellington S, Strid P, Tong VT, Woodworth K, Galang RR, Zambrano LD, et al. Characteristics of Women of Reproductive Age with Laboratory-Confirmed SARS-CoV-2 Infection by Pregnancy Status — United States, January 22–June 7, 2020. MMWR Morb Mortal Wkly Rep. 2020;
